# Use of Physical Activity Monitoring Devices by Families in Rural Communities: Qualitative Approach

**DOI:** 10.2196/10658

**Published:** 2019-02-20

**Authors:** Iryna Sharaievska, Rebecca A Battista, Jennifer Zwetsloot

**Affiliations:** 1 Department of Recreation Management and Physical Education Appalachian State University Boone, NC United States; 2 Department of Health and Exercise Science Appalachian State University Boone, NC United States

**Keywords:** motion sensors, physical activity, family, rural community

## Abstract

**Background:**

Several studies support the impact of information communication technology–based interventions to promote physical activity among youth. However, little is known on how technology can be used by the entire family to encourage healthy behavior. Previous studies showed that children and youth rely and are dependent upon the decisions and values of their caregivers when it comes to having a healthy lifestyle. Thus, the exploration of behavior and attitudes of the entire family is needed.

**Objective:**

The study aimed to explore (1) perceptions of how the use of physical activity tracking devices (Fitbit Zip) by families in rural communities influence their patterns of participation in physical activity, (2) how attitudes toward physical activity change as a result of using physical activity tracking devices as a family, and (3) what factors influence participation in physical activity among families in rural communities.

**Methods:**

A total of 11 families with 1 to 3 children of different ages (7-13 years) took part in semistructured group interviews following 2 weeks of using physical activity tracking devices (Fitbit Zip) as a family. The participants were asked to discuss their experience using the Fitbit Zip as a family, the motivation to be physically active, the changes in their pattern of participation in those activities, the level of engagement by different family members, and the factors that affected their participation. All interviews were voice-recorded with the participants’ permission and later transcribed verbatim using pseudonyms. To analyze the data, the principal investigator (IS) used open, axial, and selective coding techniques.

**Results:**

A total of 3 themes and several subthemes appeared from the data. The families in rural communities reported no or minimal changes in physical activities as a result of using physical activity tracking devices (Fitbit Zip) because of a lack of interest or an already active lifestyle. However, the attitude toward physical activity was altered. The family members reported an increased awareness of their activity level, introduced more conversations about active and healthy lifestyles, and changed their view of physical activity to a more positive one. The participants described the changes they were able to make and the constraining factors that stopped them from making further changes in their lifestyle.

**Conclusions:**

Technology might serve as a facilitator to participation in physical activity among families. Technology can motivate the change in attitude toward active recreation. As long-term changes in lifestyle require internal motivation, the change in the attitude might have a more long-lasting impact than the change in the immediate behavior. More longitudinal studies are needed to further explore long-term change in both behavior and attitude toward physical activity. Additional exploration of constraints to participation in physical activity among families is also an important area of exploration.

## Introduction

### Background

Increasing physical activity among adults and children has been a primary goal of many disciplines, government initiatives, and nonprofit organizations, including Let’s Move! Active Schools, NFL Play 60, Presidential Youth Fitness Program, Reviving Baseball in Inner Cities, and others [1,2]. Unfortunately, only 1 in 5 adults [3] and only 22% of children aged 6 to 19 years meet the physical activity guidelines [4]. There are multiple factors that may negatively influence adults’ participation in physical activities, including advanced age, low income, lack of time, low motivation, rural residency, perception of the effort needed for exercise, excessive weight or obesity, perception of poor health, and physical disability [5]. Among children and adolescents, parental support and belief in one’s ability to be active (self-efficacy), as well as walkability of the community and access to recreational spaces, were named as influential factors in physical activity participation [5,6].

### Use of Technology in Promoting Health

Contemporary technology is often seen as one of the significant constraints to participation in physical activity [7]. Technology addiction was connected to decreased exposure to the outdoors and, as a result, decrease in physical activity [7,8]. However, more and more often technology is being used to improve one’s health. For example, a study claimed that mental health information and self-help tools could be successfully delivered via the internet to rural communities where limited access to health providers and the culture of self-reliance may prevent residents from receiving needed help [9]. Moreover, multiple studies supported the effectiveness of using activity trackers and online support to improve overall health behaviors including physical activity. For example, older adults in rural communities indicated a tracking device was *easy to use* [10], whereas overweight adults showed benefits from using a virtual coach [11]. Another example of how online education programs could be used to modify health-related behaviors was in the area of sex education [12,13]. The results showed increased knowledge and improved use of healthier practices among sexually active youth [12,13]. Finally, a systematic review suggested that smartphones can be a viable tool in promoting physical activity [14], whereas a comprehensive review reported internet-based physical activity interventions can also be successful [15]. Thus, technology may in fact play an important role in changing health-related behaviors.

In the area of changing behaviors related to diet and physical activity, technology has also been shown to be a successful tool [16-19]. For example, in a study exploring diet and physical activity smartphone apps, users found these apps effective in promoting healthy eating and exercising [16]. The study participants believed the apps affected their actions, health consciousness, and self-education about nutrition and physical activity [16]. Similarly, in a study focused on the use of Fitbit trackers among female breast cancer survivors, the participants increased their physical activity, which was associated with their frequency of looking at their own data on the tracker [17]. Thus, the tracker may have been a source of motivation to alter their physical activity. There has been recent interest in using the internet and mobile phone technology to deliver automated physical activity programs to motivate adults to be more physically active [14,15,18,19]. As a result of several strategies, including the distribution of possible solutions to perceived constraints, detailing a weekly exercise plan, sharing the results with others, and providing feedback on their level of physical activity, the participants of these programs significantly increased their level of physical activity and lost a higher percent of body fat than the control group. Among an adult population with chronic obstructive pulmonary disease, it was revealed that an electronic health app was stimulating and that reaching daily physical activity goals was rewarding [19]. In addition, older adults living with chronic illness found wearable activity trackers (including Fitbit) were useful and acceptable, although participants stated they may need support with setting up the device and interpreting the data [20]. Finally, wearable activity trackers have also been used in weight loss interventions; however, the results have been mixed [21,22]. Nonetheless, tracking devices were shown to improve total step counts and percent body fat in first year medical students [23]. Although many of these interventions involve populations with various health issues (eg, overweight, obesity, and chronic illness), the tracking devices were somewhat successful in promoting physical activity. This suggests that activity tracking devices were overall a beneficial factor in behavioral change and thus could be useful in promoting physical activity among adults and youth.

### Use of Technology in Promoting Health Among Youth

The impact of SMS (short message service) text message–based interventions to promote physical activity among youth was also supported by multiple studies discussed in a review of the previous literature [24]. Out of 13 studies reviewed [24], 7 interventions resulted in improvement for physical activity, and 6 interventions resulted in improvement in sedentary behavior among youth aged 10 to 19 years. Moreover, technology-based interventions that used global positioning systems (GPS devices) as an activity (eg, geocaching and treasure hunting) were also described as more enjoyable and intrinsically motivating to youth compared with walking without technology (eg, GPS device) [25,26]. In fact, the youth had similar physical activity levels (eg, light physical activity) over the same distance (1 mile loop) in performing the required activity with the GPS device (eg, geocaching) compared with walking [27]. In other words, technology alone may be utilized to increase physical activity participation among youth. In fact, in a recent systematic review, it was suggested that physical activity trackers may be useful in promoting physical activity among this population [28]. Despite studies suggesting benefits of using technology to improve physical activity in adults with chronic disease and encourage healthy lifestyles among youth, little is known on how technology can be used by the entire family (eg, adults and youth) to promote healthy behavior that can be sustained over a long period of time.

### Families and Physical Activity

Previous studies showed that children and youth rely and are dependent upon the decisions and values of their caregivers when it comes to physical activities [6]. Studies showed that parents’ engagement in physical activity is an important predictor of children’s engagement in physical activity, both during childhood and in the later years in life [29,30]. Moreover, parents’ support and encouragement can help a child to develop physical competence and self-worth, crucial factors in long-term engagement in physical activity among children [5,31]. Although there are multiple factors that prevent families from being more active, including time and money, family structure, environment, geographical location, and others [5,6,30,32], technology could be used to lower the impact of some of these constraints by offering tips for time management or providing recommendations for physical activities that do not cost money and could be enjoyed by the entire family. This could be particularly important in rural communities where the opportunities for organized physical activities are limited. Considering that contemporary children spend more than 7 hours a day in front of a screen [33], an activity tracking device was selected as a primary factor for this study as it could be appealing to the youth interested in technology and could serve as a facilitator to participation in physical activity among families. Previous literature regarding families and physical activity was either focused on the parents’ perceptions of the benefits of and barriers to physical activity among their children [34] or revealed that the children were not very active with their parents [35]. However, a recent systematic review suggested that family-based interventions can be an effective way to improve physical activity participation among families [36]. It was suggested that the best way to improve physical activity within the family unit was to focus on family relationships and experiences (spending time together, being active as a family) rather than the benefits of being active [36].

As a result, this study focused on the families’ experience with physically active recreation and their use of activity-tracking devices. Although this study used a stand-alone physical activity tracking device (ie, Fitbit Zip), contemporary smartphones provide access to apps with similar functions (step count, distance walked, connection with others, etc), which could be used as a substitute for an activity tracking device (ie, Fitbit Zip). Thus, the objectives of this study were to explore (1) the perception of how the use of a physical activity tracking device (ie, Fitbit Zip) by families in rural communities influences their participation in physical activity, (2) how the use of a physical activity tracking device (ie, Fitbit Zip) by families in rural communities influences their attitude toward physical activity, and (3) what factors influence the participation in physical activity among families in rural communities.

## Methods

### Study Design

The study was conducted during 2014 in a small town in rural Appalachia. The study comprised 2 stages. During the first stage, 22 families with at least one child at home aged between 7 and 13 years took part in a 2-week long intervention study measuring the effect of a physical activity tracking device (Fitbit Zip) on physical activity participation in families. During those 2 weeks, each family member was asked to wear a Fitbit Zip as well as record the types of physical activities and the length of their participation in those activities into a journal. The data from the first stage of the study (eg, step counts and total physical activity levels) were presented at regional conferences and are in submission for publication. For the second stage of the study, families were asked to take part in family group interviews to reflect on their experiences using physical activity tracking devices. This paper is focused on the second qualitative part of the study and presents the results of these 11 interviews. The study protocol was approved by the university’s institutional review board. Both parents and children signed an informed consent.

### Method of Data Collection

Semistructured group interviews were conducted with each family separately in the principal investigator’s (IS) office within a 2-week period after the first stage of the study was completed. The interviews lasted between 30 min to an hour. The participants were asked to discuss their experience using the Fitbit Zip as a family, the motivation to be physically active, the changes in their pattern of participation in those activities, the level of engagement by different family members, and the factors influencing their participation. All interviews were voice-recorded with the participants’ permission and later transcribed verbatim by the principal investigator and a research assistant using pseudonyms.

### Data Analysis

Data analysis had begun as soon as several of the initial interviews were recorded and continued until the point of data saturation was reached [37]. The principal investigator used open, axial, and selective coding techniques to analyze the data [38]. This approach was selected to allow the data to speak for themselves, to *discover* and label variables and their interrelationships. After several initial interviews were conducted, the principal investigator read through the data to create labels emerging from the data. Among some of the codes that emerged during the open coding stage were “walk instead of bus, park farther, already active, and feeling accountable.” During the axial coding stage, the principal investigator identified relationships among labels emerging during the open coding stage. The codes that lacked in depth or richness were eliminated or combined to create a more general code. For example, several codes that were focused on specific activities—“walking a dog, doing things around a house, etc”—were combined into 1 category—“doing more of what they were already doing or small, everyday changes.” During the selective coding stage, the principal investigator identified core themes—perception of changes in behavior, changes in attitude, and factors influencing participation—around which the final data analysis was structured [38]. The trustworthiness of the study was enhanced by ensuring credibility, originality, resonance, and usefulness [37]. To ensure credibility, the principal investigator familiarized herself with the literature in the subject area before data analysis, stayed conscious of the depth and range of the data, as well as made sure there were strong and direct links between the gathered data and the conclusions. The principal investigator reviewed the data, codes, and themes with another qualitative researcher (BM) to ensure their logical consistency. The principal investigator also evaluated the data on the novelty and usefulness by comparing the results of the study with previous research to ensure the study provides a new insight into the phenomena. In addition, the resonance was evaluated by ensuring fullness of categories and maintaining openness toward possible meanings of data to ensure the coconstruction of new knowledge [37].

## Results

### Study Participants

A total of 11 families took part in this study. Among these families, 3 families had a father involved in the study, whereas the rest of the participants comprised mothers and their children. The families had between 1 and 3 children of different ages, with at least one child aged between 7 and 13 years. All the families self-reported as being of white background (which is a typical characteristic of the general population in rural Appalachia), and 66% of the parents had at least a Bachelor’s degree.

Several themes appeared from the data: families perceived no or minimal changes in physical activity as a result of using Fitbit Zip as a family and provided a description of small changes in their everyday activities; families discussed changes in their attitude toward physical activity as a result of using Fitbit Zip as a family; and families described factors that influenced their participation in physical activity.

### Perception of Changes in Physical Activity

#### No Changes in Physical Activity

The majority of the participants in this study reported no change in physical activity as a result of Fitbit Zip use because of their rather active lifestyle before their participation in the study or because of a lack of interest toward adopting a more active lifestyle. These findings were supported by the step count data from the first stage of the study discussed in a separate manuscript. A total of 65% (13/20) of the families who took part in the first stage of the study either did not change or decreased their daily step counts over a 2-week period, whereas 35% (7/20) increased their daily step counts.

The mother from family 6 explained:

We were already pretty active so I don’t know. [...] We get outside a fair amount, we go biking and running and stuff throughout the week outside.

Another mother (family 3) stated:

I am [...] on routines already so I don’t think I found myself looking at it.

The father from family 10 had a similar reflection:

I have a regimen and did my normal thing, I exercise 30 mins a day 5 or 6 days a week.

There was also a group of families who reported no change because of a lack of interest from the parents. For example, a boy aged 12 years (family 11) had no specific reason for why his level of physical activity did not increase. However, following his mother’s response about her busy schedule and lack of interest, he stated:

Well not much was different for me. It was kind of just daily stuff and that’s kind of how it was. [...] Nothing really changed, it’s just kind of how life is.

Although some participants lacked interest in increasing their level of physical activity, those who wanted to be more active experienced a number of constraints that stopped them from being more active.

#### Small Changes in Physically Active Recreation

There were several families who reported introducing small changes into their everyday life as a result of Fitbit Zip use. As the father from family 1 explained, he was more motivated when he could visualize his inactivity:

Yeah, it [FitBit Zip™] gave me more incentive to go do the activity because you’re looking and seeing you only have 5000 steps, I need 5000 more to go. I need to figure out something more to do today to increase my steps.

Small everyday changes mentioned by the participants included walking instead of taking a bus, parking farther away, and walking around house, etc. For example, the mother from family 6 described:

It didn’t make me exercise on a day, like if I just felt tired and felt like I wasn’t going to exercise that day, it didn’t make me plan an exercise activity, but it did make me maybe just walk around a little more and run in place while I’m making dinner.

Several families stated that although they did not introduce any new activities, they increased their level of participation in the activities in which they were already involved. Going on a longer hike or walk, practicing dancing or gymnastics more often, and other activities were listed by the participants. The mother from family 5 shared her thoughts:

I don’t think we did anything new just more often. We didn’t do anything longer just more daily than a few days a week. We tried to fit more in during the week. [...] Just having a goal.

A girl from family 4 had a similar reflection:

I’m a dancer and I’m about to start pointe after Christmas. So I would put on my pointe shoes after I had on my pajamas and practice in the living room so I could gain a couple more steps.

### Factors Influencing Participation in Physical Activity

Despite some participants lacking an interest in increasing their participation in physical activity, more participants reported an interest in living a more active lifestyle. However, many of them also experienced a number of constraints that stopped them from being more physically active. These constraints included a lack of energy, time, money, and companionship; the constraints also included environmental constraints (weather, lack of sidewalks), interest in other nonactive recreational activities, and distraction by technology.

Several participants stated that using Fitbit Zip allowed them to monitor their activities, have a healthy competition among family members, and encourage each other to be more physically active. However, despite all the benefits, many participants claimed that constraints to participation in physical activities were more significant. Among the most commonly reported constraints by both parents and children were lack of time and a busy schedule. For example, a girl from family 1 stated:

We don’t get recess anymore so we don’t really have a choice; and we have more homework and stuff so it is hard to do stuff when we are doing homework.

A boy from family 3, when asked what stopped him from being more active, shared a similar experience:

School, 6 and a half hours, no going outside.

The mother from family 5 summarized her daughter’s experiences:

She has a lot of stuff going on. She is in a play and has rehearsal 3 times a week and swimming twice a week. Just having to be somewhere. Schedule.

Environmental constraints, including weather and lack of sidewalks, were also mentioned by the participants. The mother from family 3 explained:

I miss sidewalks since moving up here, you can’t just go out and do in a safe way. I definitely have to coordinate with what they are doing. I think just scheduling makes it difficult to work it in or you have to get really creative.

Several participants reported distraction by technology as one of the constraints to physical activity participation among children. For example, the father from family 1 stated “Instagram” when asked about constraining factors, whereas his son confessed, “I’m glued to the Wii.” Thus, although technology such as Fitbit Zip may encourage and motivate slight changes in physical activity participation among families, there are multiple factors that may be more powerful constraints when it comes to leading a more active lifestyle.

### Change in the Attitude Toward Physical Activity

#### Increased Awareness and Accountability

The majority of participants in this study reported a change in their attitude toward physical activity. They reported becoming more aware of what it takes to have an active lifestyle, they reported increased conversations in the family about health and physical activities, and they also reported a change in their view of physical activity to a more positive one. For example, many participants were surprised by how active or inactive their everyday activities were. The mother from family 6 explained:

It made you a little more aware maybe when you thought you got enough exercise but maybe you didn’t quite. [...] I think it did make me more aware and how even just doing some little things, like we live near the [grocery store] and how walking to the [grocery store] could add, little bit more walking home from school, how that can give you some more steps.

In many cases, this increased level of awareness motivated study participants to be more active during their recreational and everyday activities. The mother from family 3 described her son’s increase in motivation as a result of using Fitbit Zip:

I think more awareness. He [son] was extra aware and made more of an effort like “oh I need to feed the FitBit Zip™”so he was making an extra effort. I could see that when he actually could see it, he was taking extra time to find ways, so yeah I noticed that behavior about him.

Similarly, the mother from family 5 shared her own increase in motivation to be more physically active as a result of Fitbit Zip use:

I would try to be more active because before I would think 3 days a week was good. But thinking about the 10,000 each day made me want to do more.

#### More Conversations About Active Recreation

In addition to an increased awareness, the Fitbit Zip use provoked more conversations about an active lifestyle and health in general among family members. As the father from family 10 described:

In the evening at least we talked about the steps and what they were doing and what they did for PE and things like that.

His daughter added:

At dinner we would usually look at our fitbits and all say how many steps we did.

Comparing the number of steps among different family members allowed families to be more intentional in planning physical activity:

They were constantly looking and seeing how many steps they had and what they were going to do.

Mother, family 1

Moreover, families enjoyed the friendly competition with each other and celebrated the achievement of the winning family member, which further initiated conversations about active recreation. The mother from family 3 explained:

I think it was cool that we were thinking about it and checking on each other. [...] I think we kind of talked about it and looked at it, maybe twice a day or so. [...] It is kind of interesting because he [son] is ultra-competitive with it [...], and it was kind of neat to celebrate with him and be like how did you get that many? What did you do today to get there? So I think for me as a parent it was fun to see him have this accomplishment and know that there were many days that he had more than I had.

#### Physical Activity as a Benefit

Finally, the participants reported that after using the physical activity tracking device as a family, their view of physical activity was changed to a more positive one. As a result of the participation in the study, many participants changed their perception of physical activity from inconvenient and burdensome to beneficial. For example, walking the dog, parking farther away, and getting something from another room were now the activities that family members did not mind doing. For example, a mother from one of the least active families stated:

I had a better attitude about being more physical, like instead of being annoyed I had to park further away, I would be like that’s ok. This is good for me.

A father described how he was using the Fitbit Zip to motivate his children to walk the dog together. The son from family 10 described how being active was no longer perceived as a burden by him and his sibling but rather as an opportunity to earn more steps:

One night someone was like “will you go get such and such for me” and no one would get off the couch, [...] and when she said that or anyone said that to me, I would be like “I’ll do it.” I got up and got up straight to do it.

## Discussion

### Principal Findings

Overall, the major finding from this study suggests a physical activity tracking device may not immediately impact total physical activity but may provide an awareness of total physical activity participation among families. The awareness or perception regarding how active or inactive they are as individuals or as a family may eventually lead to actions to become more active despite some commonly discussed constraints.

The findings in this study highlight several important issues related to increasing the level of physical activity among children previously discussed in the literature. First, adults play a crucial role when it comes to the health and active lifestyle of the entire family [31]. As our data suggested, parents who were interested in increasing physically active recreation encouraged their children’s participation, introduced discussions about experiences, and celebrated their children’s achievements. Unfortunately, many of them also reported multiple constraints that prevented the family’s participation in physical activity, including a lack of energy, time, money, companionship; the constraints also included environmental constraints (weather, lack of sidewalks), interest in other nonactive recreational activities, and distraction by technology. Second, to increase participation in physical activity among children, it is important to make it fun yet still be challenging [31]. Our participants reflected on the enjoyment from the experience of using Fitbit Zip and friendly competition among family members. Thanks to the evening discussions about the step counts every family member obtained during the day, many children were excited to compete with their siblings and parents and, as a result, found the process of being active to be fun. Finally, as previous studies described that the sense of mastery and self-efficacy often encourages children’s engagement in physical activities [31,39], physical activity tracking devices could be used to enhance those emotions among youth. For example, having a set goal and being able to observe (on the activity tracking device) how one’s actions can allow the individual to achieve that goal might develop a sense of mastery and self-efficacy among youth. Goal setting and reinforcement were among the suggested ways to increase family-based physical activity discussed in a recent meta-analysis review [36]. The perception of a great effort needed for exercise was mentioned in previous research as one of the constraints for physical activity among adults [5]. The users of activity tracking devices in this study mentioned how the small changes in their everyday routine and increased participation in activities they already enjoyed allowed the participants to increase their step count in an easy way with little or no preparation. Thus, the use of the activity tracking device could assist in changing the perception of physical activity from physical activity requiring great effort to it being simpler and more manageable.

The important message from this study is the relationships between technology and physically active recreation. Although technology is often viewed as a constraint to physical activity [7], it might also be used as a facilitator [39]. Moreover, as the results of this study suggest, a physical activity tracking device can motivate the change in attitude toward active recreation. As intrinsic motivation is often viewed as an important part of long-term change in behavior [40], the change in attitude and perception of physical activity and a healthy lifestyle overall might have a valuable impact on the future of physical activity among families.

### Limitations

Although the study brings attention to some important areas in the research on physically active recreation, it also has several limitations. First, because of the self-selection process, a majority of the participants who agreed to participate in the study were already predominantly active and healthy residents of the community. Second, the engagement of families with physical activity tracking devices (Fitbit Zip) was rather short (2 weeks), and the novelty and excitement from this experience might decrease with time [41]. In future studies, we recommend to allow participants a longer period of engagement, as well as to ensure that the interviews are conducted soon after participation as well as several months after the experience ends. Third, the data were analyzed by only 1 of the authors. To address this limitation, the authors reviewed the data, codes, and themes with other qualitative researchers to ensure their logical consistency. Despite these limitations, the study provided a novel perspective on family units and their use of technology to modify health-related behavior such as physical activity. In addition, the study used a qualitative approach to gauge a deep understanding of the perception of the experience with technology and physical activity as a family.

### Conclusions

Family-based physical activity interventions may be an effective way to improve the physical activity for both adults and children. Our results suggest that introducing simple everyday activities into family routines may encourage parents and their children to increase their overall physical activity level. Moreover, it may lead to a changed perception of physical activities from difficult and time-consuming to easy and enjoyable. Our results also suggest that facilitating the engagement of the entire family with a physical activity intervention may encourage more discussions about active recreation and the ways to achieve it. Such discussions, healthy competition, and celebrations of achievements among family members may further promote a healthy and active lifestyle among the family as a unit. Finally, the use of activity tracking devices by families can allow both parents and children to be aware of the levels of physical activities among family members in their family unit, as well as observe what activities and strategies can be the most helpful in achieving their goals for physical activities.

There are several implications for future research in this area. Due to an increased presence of technology in the lives of families, we suggest more studies focused on the use of technology to promote physical activities among children and families are needed. More specifically, further studies should explore whether the latest technology can be used as a successful facilitator for physical activities among families. Family is a complex system, and a change in one area of life of the family (eg, employment of parents) may lead to changes in other areas of life (eg, use of technology or physical activity participation). Moreover, families may have various structures and go through various stages in life, as well as represent individuals of diverse backgrounds. A more thorough understanding of the barriers of those with diverse backgrounds may assist in developing more effective family-based physical activity interventions. It is also important to utilize different methods to evaluate the family’s physical activity. For example, measuring the total physical activity of each family member and comparing the perception that families have regarding their personal and children’s physical activity may be a critical component. Thus, it is important to continue both qualitative and quantitative exploration of this area of study to a produce theoretical foundation and empirical results that could further inform the work of practitioners working with families.

## Abbreviations

GPS: global positioning system
SMS: short message service

## References

[1] National Football League.

[2] Let's Move!.

[3] (2014). Centers for Disease Control and Prevention 2014.

[4] (2016). National Physical Activity Plan.

[5] Healthy People 2020.

[6] Van Der Horst K, Paw MJ, Twisk JW, Van Mechelen W (2007). A brief review on correlates of physical activity and sedentariness in youth. Med Sci Sports Exerc.

[7] Louv R (2008). Last child in the woods: Saving our children from nature-deficit disorder.

[8] Watkins SC (2010). The Young and the Digital: What the Migration to Social-Network Sites, Games, and Anytime, Anywhere Media Means for Our Future.

[9] Griffiths KM, Christensen H (2007). Internet-based mental health programs: a powerful tool in the rural medical kit. Aust J Rural Health.

[10] Batsis JA, Naslund JA, Gill LE, Masutani RK, Agarwal N, Bartels SJ (2016). Use of a wearable activity device in rural older obese adults: a pilot study. Gerontol Geriatr Med.

[11] Watson A, Bickmore T, Cange A, Kulshreshtha A, Kvedar J (2012). An internet-based virtual coach to promote physical activity adherence in overweight adults: randomized controlled trial. J Med Internet Res.

[12] Chong A, Gonzalez-Navarro M, Karlan D, Valdivia M (2017). National Bureau of Economic Research.

[13] Simon L, Daneback K (2013). Adolescents' use of the internet for sex education: a thematic and critical review of the literature. Int J Sex Health.

[14] Bort-Roig J, Gilson ND, Puig-Ribera A, Contreras RS, Trost SG (2014). Measuring and influencing physical activity with smartphone technology: a systematic review. Sports Med.

[15] Joseph RP, Durant NH, Benitez TJ, Pekmezi DW (2014). Internet-based physical activity interventions. Am J Lifestyle Med.

[16] Wang Q, Egelandsdal B, Amdam GV, Almli VL, Oostindjer M (2016). Diet and physical activity apps: perceived effectiveness by app users. JMIR Mhealth Uhealth.

[17] Hartman SJ, Nelson SH, Weiner LS (2018). Patterns of Fitbit use and activity levels throughout a physical activity intervention: exploratory analysis from a randomized controlled trial. JMIR Mhealth Uhealth.

[18] Hurling R, Catt M, De Boni M, Fairley BW, Hurst T, Murray P, Richardson A, Sodhi JS (2007). Using internet and mobile phone technology to deliver an automated physical activity program: randomized controlled trial. J Med Internet Res.

[19] Vorrink SN, Kort HS, Troosters T, Lammers JW (2016). A mobile phone app to stimulate daily physical activity in patients with chronic obstructive pulmonary disease: development, feasibility, and pilot studies. JMIR Mhealth Uhealth.

[20] Mercer K, Giangregorio L, Schneider E, Chilana P, Li M, Grindrod K (2016). Acceptance of Commercially Available Wearable Activity Trackers Among Adults Aged Over 50 and With Chronic Illness: A Mixed-Methods Evaluation. JMIR Mhealth Uhealth.

[21] Jakicic JM, Davis KK, Rogers RJ, King WC, Marcus MD, Helsel D, Rickman AD, Wahed AS, Belle SH (2016). Effect of wearable technology combined with a lifestyle intervention on long-term weight loss: the IDEA randomized clinical trial. JAMA.

[22] Wang JB, Cadmus-Bertram L, Natarajan LA, White M, Madanat H, Nichols JF, Ayala GX, Pierce JP (2015). Wearable sensor/device (Fitbit One) and SMS text-messaging prompts to increase physical activity in overweight and obese adults: a randomized controlled trial. Telemed J E Health.

[23] DiFrancisco-Donoghue J, Jung MK, Stangle A, Werner WG, Zwibel H, Happel P, Balentine J (2018). Utilizing wearable technology to increase physical activity in future physicians: a randomized trial. Prev Med Rep.

[24] Ludwig K, Arthur R, Sculthorpe N, Fountain H, Buchan DS (2018). Text messaging interventions for improvement in physical activity and sedentary behavior in youth: systematic review. JMIR Mhealth Uhealth.

[25] Battista RA, West ST, Houge Mackenzie S, Son JS (2016). Is this exercise? No, it’s geocaching! Exploring factors related to aspects of geocaching participation. J Park Recreat Admi.

[26] Boulos MN, Yang SP (2013). Exergames for health and fitness: the roles of GPS and geosocial apps. Int J Health Geogr.

[27] Battista RA, West ST (2018). The use of geocaching as a form of physical activity in youth. Am J Health Educ.

[28] Ridgers ND, McNarry MA, Mackintosh KA (2016). Feasibility and Effectiveness of using wearable activity trackers in youth: a systematic review. JMIR Mhealth Uhealth.

[29] Dunton GF, Liao Y, Almanza E, Jerrett M, Spruijt-Metz D, Chou CP, Pentz MA (2012). Joint physical activity and sedentary behavior in parent-child pairs. Med Sci Sports Exerc.

[30] Mulhall P, Reis J, Begum S (2011). Early adolescent participation in physical activity: correlates with individual and family characteristics. J Phys Act Health.

[31] Weiss MR (2000). Motivating kids in physical activity. Pres Counc Phys Fit Sports Res Dig.

[32] Trussell DE, Shaw SM (2009). Changing family life in the rural context: women's perspectives of family leisure on the farm. Leis Sci.

[33] US Department of Health & Human Services.

[34] Lakes KD, Abdullah MM, Youssef J, Donnelly JH, Taylor-Lucas C, Goldberg WA, Cooper D, Radom-Aizik S (2017). Assessing parent perceptions of physical activity in families of toddlers with neurodevelopmental disorders: the parent perceptions of physical activity scale (PPPAS). Pediatr Exerc Sci.

[35] Jago R, Sebire SJ, Wood L, Pool L, Zahra J, Thompson JL, Lawlor DA (2014). Associations between objectively assessed child and parental physical activity: a cross-sectional study of families with 5-6 year old children. BMC Public Health.

[36] Brown HE, Atkin AJ, Panter J, Wong G, Chinapaw MJ, van Sluijs EM (2016). Family-based interventions to increase physical activity in children: a systematic review, meta-analysis and realist synthesis. Obes Rev.

[37] Charmaz K (2016). Constructing Grounded Theory: A Practical Guide Through Qualitative Analysis.

[38] Strauss A (1987). Qualitative Analysis for Social Scientists.

[39] Raymore LA (2017). Facilitators to Leisure. J Leis Res.

[40] Frederick CM, Ryan RM (1993). Differences in motivation for sport and exercise and their relations with participation and mental health. J Sport Behav.

[41] Tassey G, Carayannis EG (2013). Technology life cycles. Encyclopedia of Creativity, Invention, Innovation and Entrepreneurship.

